# A novel approach based on the ultrasonic-assisted microwave method for the efficient synthesis of Sc-MOF@SiO_2_ core/shell nanostructures for H_2_S gas adsorption: A controllable systematic study for a green future

**DOI:** 10.3389/fchem.2022.956104

**Published:** 2022-10-10

**Authors:** Khursheed Muzammil, Reena Solanki, Ayad F. Alkaim, Rosario Mireya Romero Parra, Holya A. Lafta, Abduladheem Turki Jalil, Reena Gupta, Ali Thaeer Hammid, Yasser Fakri Mustafa

**Affiliations:** ^1^ Department of Public Health, College of Applied Medical Sciences, Khamis Mushait Campus, King Khalid University, Abha, Saudi; ^2^ Department of Chemistry, Dr. A. P. J. Abdul Kalam University, Indore, Madhya Pradesh, India; ^3^ Chemistry Department College of Science for Women University of Babylon, Hillah, Iraq; ^4^ Universidad Continental Lima, Lima, Perú; ^5^ Department of Pharmacy, Al Nisour University College, Baghdad, Iraq; ^6^ Medical Laboratories Techniques Department, Al Mustaqbal University College, Babylon, Iraq; ^7^ Institute of Pharmaceutical Research, GLA University, Mathura, India; ^8^ Computer Engineering Techniques Department, Faculty of Information Technology, Imam Ja’afar Al Sadiq University, Baghdad, Iraq; ^9^ Department of Pharmaceutical Chemistry, College of Pharmacy, University of Mosul, Mosul, Iraq

**Keywords:** sc-MOF@SiO2, core/shell nanostructures, ultrasonic assisted microwave, H2S gas, adsorption process

## Abstract

In this work, for the first time, novel Sc-MOF@SiO_2_ core/shell nanostructures have been synthesized under the optimal conditions of ultrasonic-assisted microwave routes. The final products showed small particle size distributions with homogeneous morphology (SEM results), high thermal stability (TG curve), high surface area (BET adsorption/desorption techniques), and significant porosity (BJH method). The final nanostructures of Sc-MOF@SiO_2_ core/shell with such distinct properties were used as a new compound for H_2_S adsorption. It was used with the systematic investigation based on a 2^K−1^ factorial design, which showed high-performance adsorption of about 5 mmol/g for these novel adsorbents; the optimal experimental conditions included pressure, 1.5 bar; contact time, 20 min; and temperature, 20°C. This study and its results promise a green future for the potential control of gas pollutants.

## 1 Introduction

Recently, the applications of metal-organic frameworks (MOFs) have received special attention due to their desirable properties (O'Neill et al., 2010; Yang et al., 2021; Chen et al., 2022). These compounds, which consist of various metals and linkers with their mechanical strength, thermal stability, and high specific surface, have many applications in industry, environment, and medicine (Ding et al., 2019; Wang and Astruc, 2019; Shyngys et al., 2021).

Due to the spread of air pollutants, controlling and reducing them is a necessity. Gaseous pollutants are an important group of pollutants that have affected the environment, humans, and other animals (Cesur et al., 2017). One of the most critical types of gaseous pollutants is sulfide gas, such as H_2_S molecules. This compound, which has increased with population, has adverse environmental effects; therefore, small amounts are dangerous (Lantto and Mizsei, 1991).

Various methods for controlling and trapping gaseous pollutants have been studied, including catalytic processes, absorption, and adsorption procedures (Perera et al., 2012; Ma et al., 2018; Zhang et al., 2020). Adsorption is an inexpensive, controllable, simple, and green process that has been highlighted compared to other methods. Previous studies have also confirmed the importance of the adsorption procedure in comparison with other classical methods (Li et al., 2020). MOFs with desirable physicochemical properties are one of the novel candidates for the adsorption process (Krishna et al., 2011; Xi et al., 2022). These compounds have high surface area, significant porosity, and desirable mechanical features, which are attractive for the adsorption of various compounds (Krishna et al., 2010; Zhu et al., 2022). These novel crystalline compounds, which consist of metal nodes and organic ligands, have some structural flexibility and textural properties that make them a very effective class of compounds for potential adsorption processes.

On the other hand, the type of synthesis method also has a great effect on the amount of gas adsorption. MOFs are synthesized in a variety of ways, including ultrasonic, microwave, sol–gel, and co-precipitation methods (Rivera-Muñoz and Huirache-Acuña, 2010; Guo et al., 2022). The use of novel methods such as ultrasonic and microwave compared to conventional methods not only synthesizes samples in short time but also affects the physicochemical properties of the final compounds (Qiu et al., 2014; González et al., 2021).

One of the effective factors in increasing the functional efficiency of MOF nanostructures on gas adsorption is a systematic process study. Among the parameters affecting the efficiency of gas adsorption, temperature, time contact, and pressure are important factors (Alhamami et al., 2014; Guo et al., 2021). It is critical to systematically study the effect of these parameters on gas adsorption. The use of conventional systematic study increases the number of experiments significantly, which results in a long testing process (Pu et al., 2021). Recently, the use of a 2^k−1^ factorial design has been considered for designing experiments, which results in the production of novel products with distinctive features (Yu and Sepehrnoori, 2014).

Although MOF nanostructures have distinct properties compared to other compounds, increasing their specific surface area for functional potential is a major challenge (Falcaro et al., 2014; Rubio-Martinez et al., 2017). Recently, the synthesis of core/shell nanostructures has been given special attention in order to increase the specific surface area and stability of the product (Liu and Tang, 2013; Liu et al., 2022). Silica is one of the stable substrates that in the form of a shell can affect the specific surface of the product (Kalambate et al., 2019).

In this study, for the first time, Sc-MOF@SiO_2_ core/shell nanostructures were synthesized by combining Sc-MOF nanostructures and SiO_2_ shell powders, and their properties were characterized using scanning electron microscopy (SEM), energy-dispersive spectrometry (EDS) mapping analysis, Fourier transform-infrared (FT-IR) spectroscopy, thermogravimetric analysis (TGA), and BET surface area technique. Finally, adsorption studies were developed systematically for H_2_S gas adsorption with a 2^k−1^ factorial design.

## 2 Materials and methods

### 2.1 Materials

Scandium 3) nitrate hexahydrate with MW of 248.99 g/mol and purity of 99.90 (Sigma-Aldrich, Steinheim, Germany), 2,6 pyridine dicarboxylic acid with MW of 167.12 g/mol and purity of 99.80% (Sigma-Aldrich, Steinheim, Germany), silicon dioxide substrate with MW of 60.08 g/mol and purity of 99.80% (Sigma-Aldrich, Steinheim, Germany), and H_2_S capsule with purity of 98.98% (Sigma-Aldrich, Steinheim, Germany) were prepared without any purification. Deionized water obtained by the Millipore Milli-Q system (Darmstadt, Germany) was used in all experiments.

### 2.2 Characterization of the products

The particle size distribution and morphology of the products were determined using a scanning electron microscope (SEM) (EVO 10, Carl Zeiss AG, Jena, Germany). FTIR spectroscopy was performed on a Nicolet-6700 FTIR spectrometer with a wavenumber range of 400–4,100 cm^−1^. TGA was measured using a Netzsch Thermoanalyzer STA 409 in an Ar atmosphere at a heating rate of 5°C/min. The BET surface areas of Sc-MOF@SiO2 core shell nanostructures were determined using a Micromeritics TriStar II 3020 analyzer.

### 2.3 Synthesis of Sc-MOF nanostructures

In a typical microwave-assisted synthesis, 0.0235 g of Sc (NO3)_3_ (0.2 mmol) and 0.0722 g of pyridine-2,6 dicarboxylic acid were dissolved in 40 ml of double-distilled water, and the mixture was stirred for approximately 35 min at 70°C. Then, the resultant solutions were transferred to the microwave device and placed under optimal microwave radiation with a power of 300 W for 40 min at an ambient temperature.

### 2.4 Synthesis of Sc-MOF@SiO_2_ core/shell nanostructures

The synthesis of Sc-MOF@SiO_2_ core/shell nanostructures using the ultrasonic-assisted microwave method is as follows: the mixture obtained in the previous stage (2.3) was added to 0.453 mg of SiO_2_ nanostructures. The compound is placed in the ultrasonic device with a frequency of 21 Hz and subjected to ultrasound irradiation with a power of 20 W for 40 min at a temperature of 33°C. After cooling to room temperature, the nanostructures were isolated by washing with distilled water.

## 3 Results and discussion

### 3.1 Characterization of nanostructures

#### 3.1.1 Morphology and particle size distribution

**FIGURE 1:**
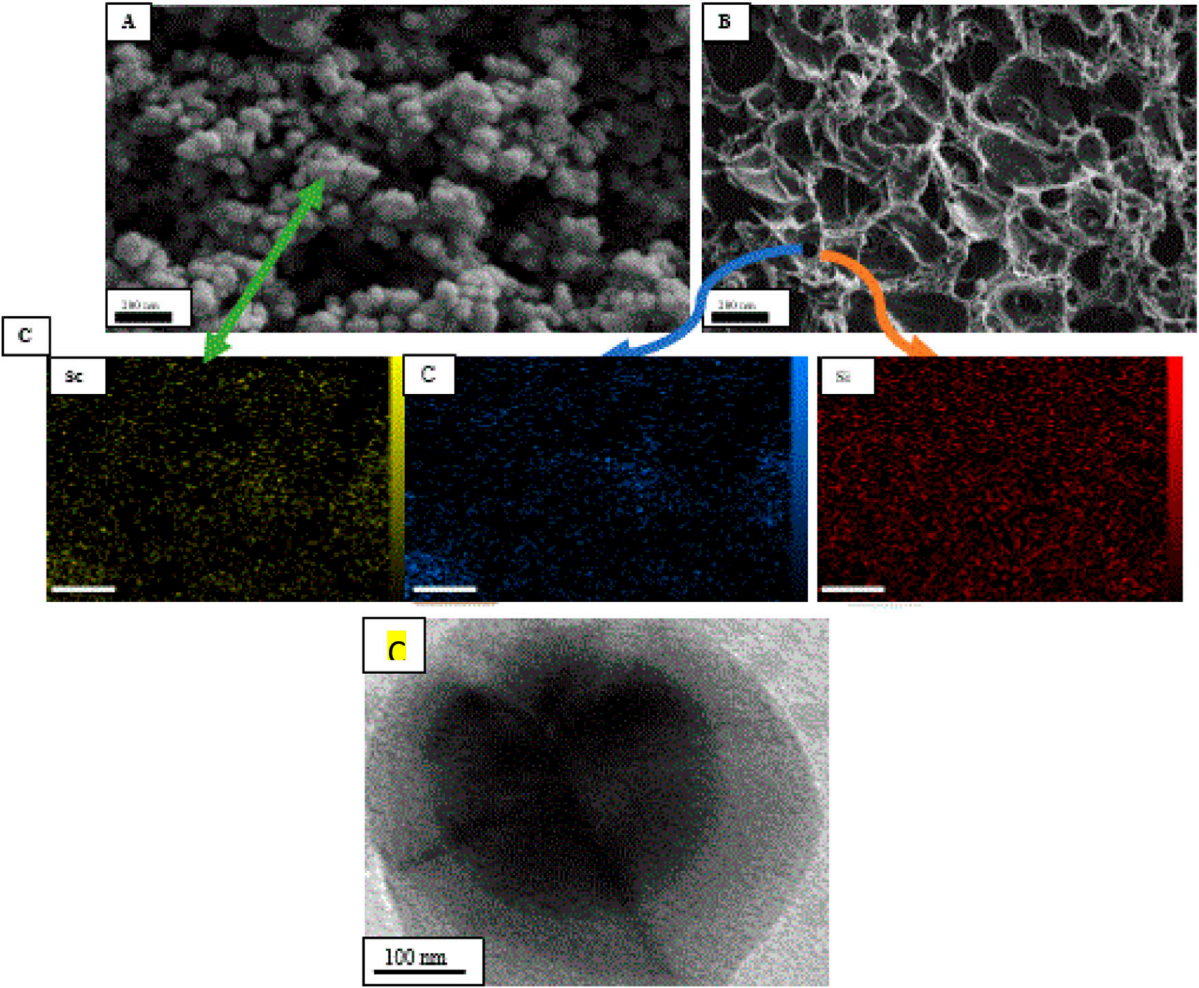
SEM images **(A)** Sc-MOF and **(B)** Sc-MOF@SiO_2_) with **(C)** EDS elemental analysis for core (Sc and C) and shell structures (Si) and **(D)**TEM image of Sc-MOF@SiO_2_ core/shell nanostructures.

**FIGURE 2:**
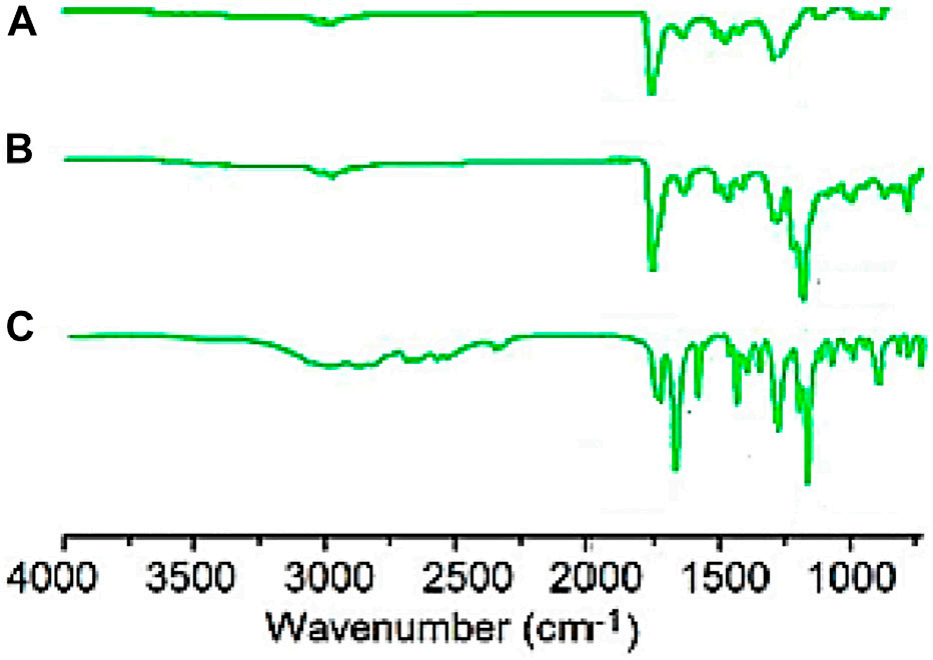
FTIR spectra of **(A)** SiO_2_, **(B)** Sc-MOF, and **(C)** Sc-MOF@SiO_2_ core/shell nanostructures.

The mean particle size distribution along with the morphology of Sc-MOF nanostructures and the Sc-MOF@SiO_2_ core/shell nanostructures are shown in Figure 1A,B. Accordingly, the mean particle size distribution of the Sc-MOF was distributed in the nanometric range (diameter less than 100 nm). According to Figure 1B, the Sc-MOF nanoparticles are agglomerated in the core/shell network of the Sc-MOF@SiO_2_ nanostructures with a uniform distribution. It can be seen that the morphology of Sc-MOF was slightly changed in the final structures, which can be related to the effects of ultrasonic-assisted microwave methods. In order to ensure the presence of the characteristic elements in the final structures, the EDS elemental with mapping analysis has been used, and, as seen in Figure 2, the related elements are displayed in the final product. As an important result, the synthesis of Sc-MOF@SiO_2_ core/shell nanostructures by the ultrasonic-assisted microwave method was confirmed. Also, in order to ensure that the core and shell nanostructures are formed, TEM images of Sc-MOF@SiO_2_ core/shell nanostructures were taken. As shown in Figure 1D, there is a clear distinction between the core (Sc-MOF) and shell (SiO_2_) structures. As an important result, the formation of MOF@SiO_2_ core/shell nanostructures with a homogeneous morphology improves the functional potential of these nanostructures in the field of gas adsorption.

#### 3.1.2 Suggested structures

**FIGURE 3:**
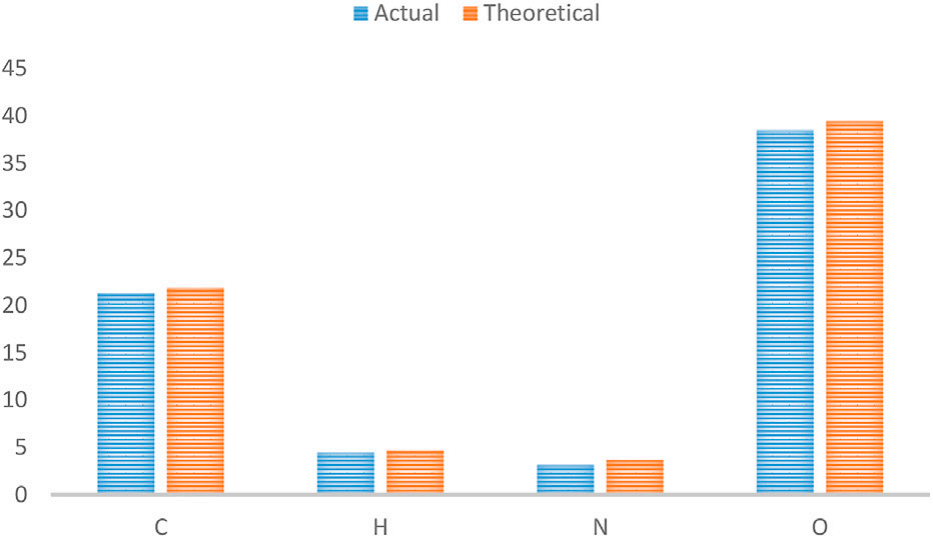
CHNSO/analysis for Sc-MOF@SiO_2_ core/shell nanostructures synthesized using the ultrasonic-assisted microwave method.

**FIGURE 4:**
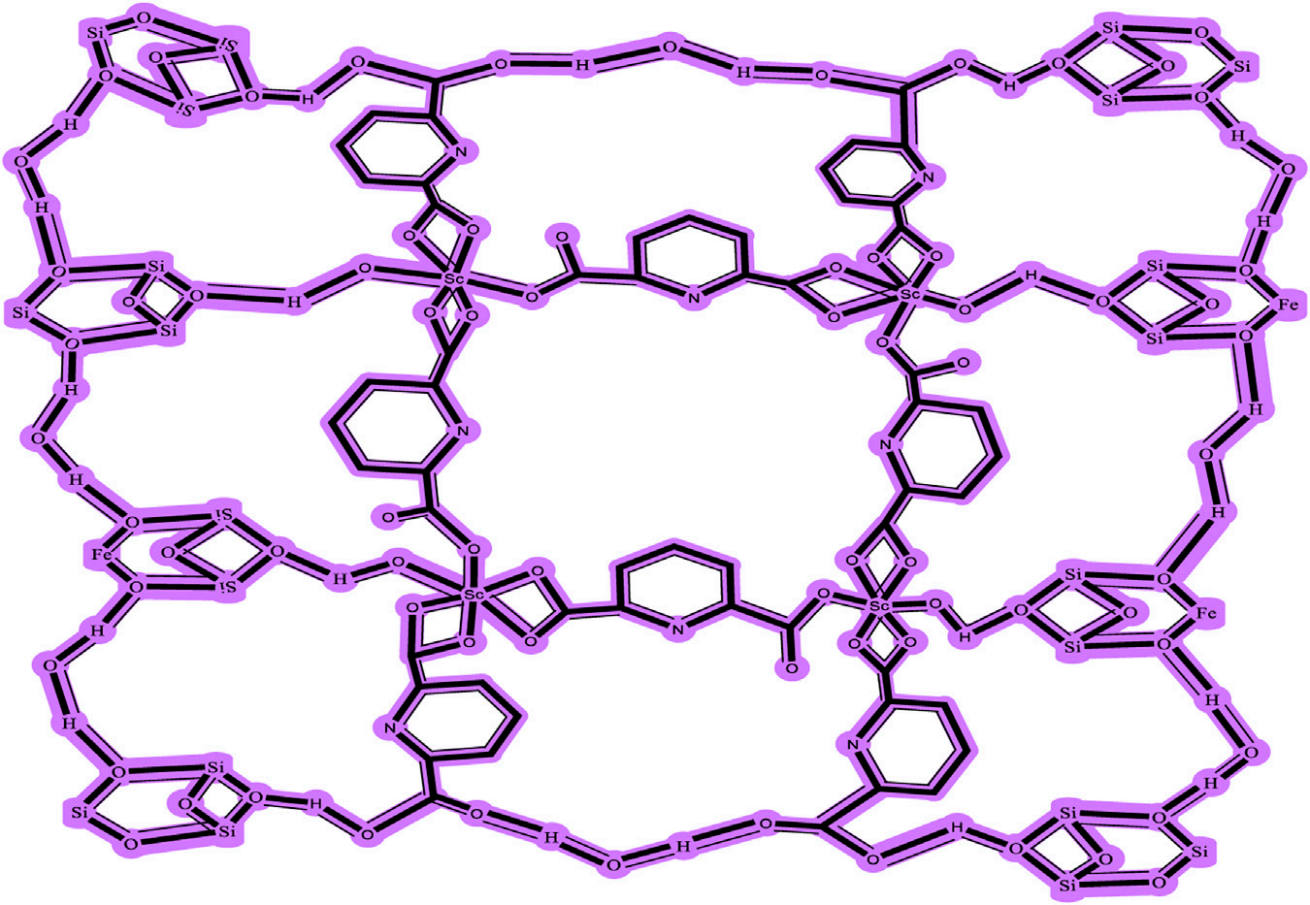
Suggested formula for Sc-MOF@SiO_2_ core/shell nanostructures synthesized using the ultrasonic-assisted microwave method.

Figure 2 depicts the FTIR spectra of SiO_2_ structures (A), Sc-MOF (B), and Sc-MOF@SiO_2_ core/shell nanostructures (C). According to the FTIR spectra of Sc-MOF, the absorption peaks at 3,070 cm^−1^ may be related to the coordinated solvent in the products. The peaks near 2,800 cm^−1^ may be attributed to the aromatic CH groups. The strong bands at 1,360 and 1,490 cm^−1^ correspond to the asymmetric and symmetric stretching peaks of COO groups, respectively (Rojas et al., 2014). The absorption bands at 800 cm^−1^ are assigned to C–H bonds. For both SiO_2_ and Sc-MOF nanostructures, the peaks near 2,500–1,500 cm^−1^ can be attributed to the Si–O and Sc–O bonds, respectively (Han et al., 2015; Zhang et al., 2022). According to Figure 2C, the characteristic peaks of SiO_2_ and Sc-MOF can be seen in the FTIR spectrum of Sc-MOF@SiO_2_ core/shell nanostructures. Figure 3 shows the CHNS/O elemental analysis of Sc-MOF@SiO_2_ core/shell nanostructures. According to this figure, the amounts of related elements of C, H, N, and O are distributed well. As an important result, based on the FTIR spectra of samples, different linker configurations (Watanabe et al., 2006; Deng et al., 2019), and also CHNS/O analysis, the suggested structures for Sc-MOF@SiO_2_ core/shell nanostructures are shown in Figure 4.

#### 3.1.3 Thermal stability and surface area

**FIGURE 5:**
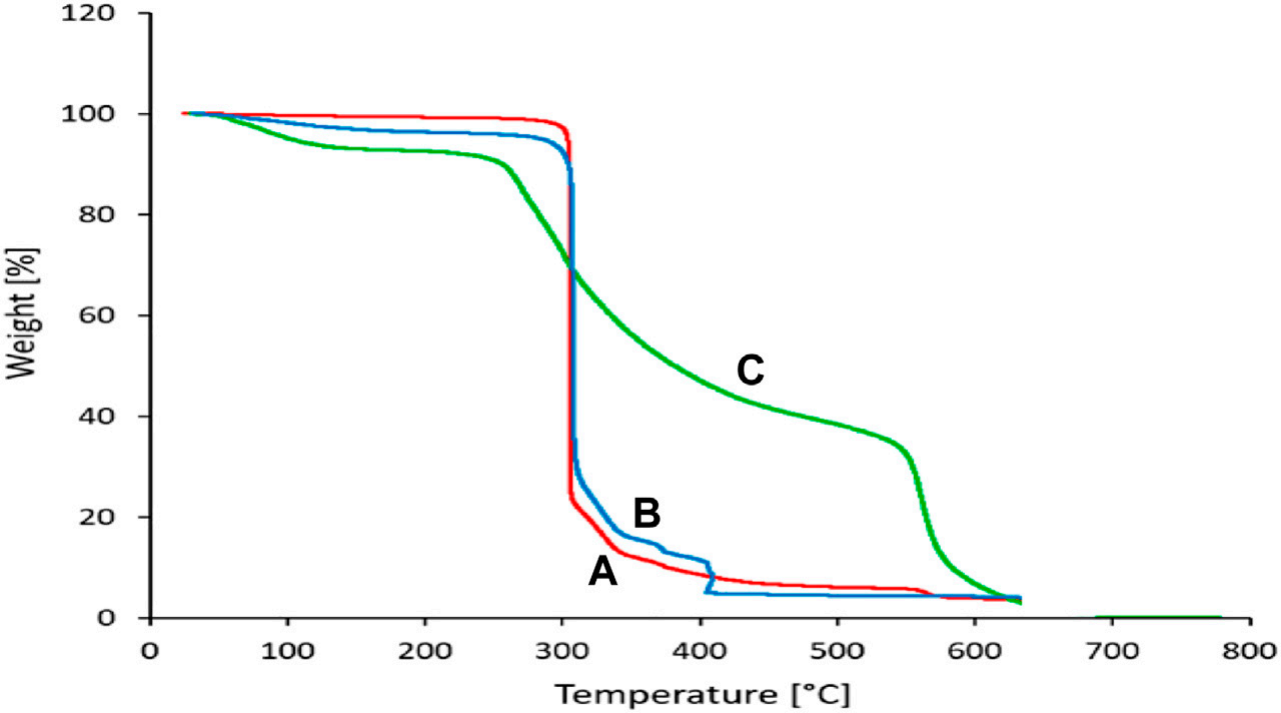
Thermal stability of **(A)**: SiO_2_ powder, **(B)**: Sc-MOFs, and **(C)**: Sc-MOF@SiO2 core/shell nanostructures.

The thermal stability of siO_2_, Sc-MOF, and Sc-MOF@SiO_2_ core/shell nanostructures is shown in Figure 5. By comparing these peaks, the Sc-MOF@SiO_2_ core/shell nanostructures have a high thermal stability (328°C) compared to the SiO_2_ nanoparticles (264°C) and Sc-MOF nanostructures (292°C). As an important result, the thermal stability of the Sc-MOF@SiO_2_ core/shell nanostructures developed in this study is greatly increased compared to similar samples (Nazir et al., 2021; Zhao et al., 2021). This can be related to the choice of structure type and the ultrasonic-assisted microwave route. The high thermal stability can provide a significant possibility for the application of this compound in different areas, such as novel adsorption.

**FIGURE 6:**
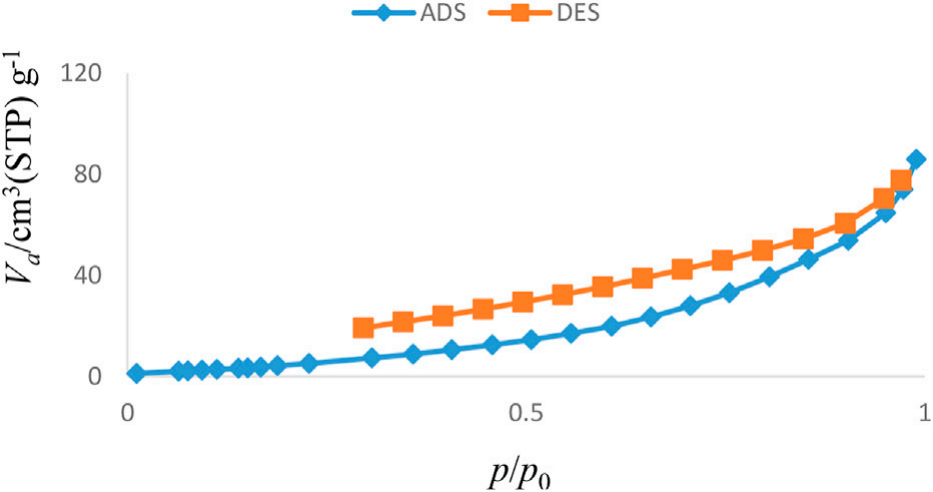
N_2_ adsorption/desorption isotherms of Sc-MOF@SiO2 core/shell nanostructures.

**FIGURE 7:**
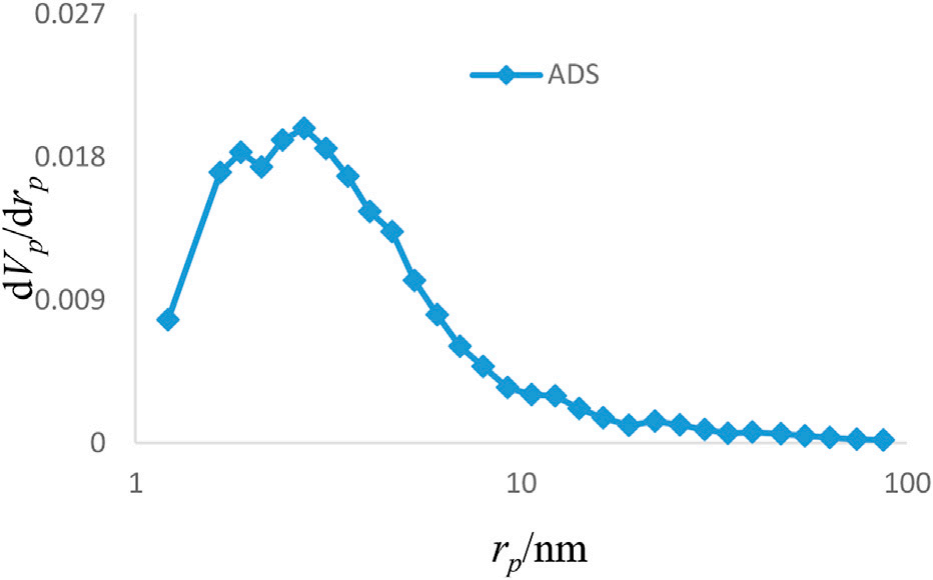
BJH pore size distribution for Sc-MOF@SiO_2_ core shell nanostructures.

The results of various analyses showed that the Sc-MOF@SiO_2_ core–shell nanostructures have better physicochemical properties than pure Sc-MOF and SiO_2_ powders. Therefore, these compounds were selected as new products for further applications. Figure 6 shows the adsorption/desorption isotherms of the Sc-MOF@SiO_2_ core/shell nanostructures synthesized by the ultrasonic-assisted microwave method. Based on this isotherm, the adsorption/desorption behaviors of the samples are similar to the second series of classical isotherms, which confirms the mesoporous behavior (size distribution between 2 and 50 nm) for the final sample (Ebadi et al., 2009). Also, based on the BET results, Sc-MOF@SiO_2_ core/shell nanostructures have a surface area of about 3,700 m^2^/g. In order to correlate between adsorption isotherms and particle size distributions, the BJH method has been used. As shown in Figure 7, the size of the pore distribution was more than 2 nm, which confirms the significant porosity of Sc-MOF@SiO_2_ core/shell nanostructures with mesopore size distributions. As an important result, the synthesis of samples with high porosity provides the applicable potential for adsorption procedures.

### 3.2 H_2_S gas adsorption

#### 3.2.1 Experimental design

Sc-MOF@SiO2 core/shell nanostructures have been selected as a new option for gas adsorption due to their desirable properties such as narrow particle size distribution, high thermal stability, remarkable surface area, and significant porosity. In order to measure the amount of H_2_S gas adsorption by these novel nanostructures, a volumetric method has been used. This method was carried out based on previous studies (Roy et al., 2017). In order to systematically design the process and investigate the effective experimental parameters on H_2_S adsorption, the 2^k−1^ method has been used. Effective parameters included pressure (A), time contact (B), and temperature (C). The values of each of these parameters and the results of the H_2_S gas adsorption are presented in Table 1. Also, the distributions of experiments with two repetitions are presented in Table 2.

**TABLE 1:** 2^k−1^ factorial design for H_2_S gas adsorption studied by Sc-MOF@SiO2 core/shell nanostructures.

Level	Coded level	Uncoded level		
		Pressure (bar)	Time contact (min)	Temperature (°C)
Low	−1	0.5	2	20
Center	0	1	4	25
High	+1	1.5	6	30
**Coded formula:** $\frac{x-\frac{x\;\left(high\right)+x\left(low\right)}{2}}{\frac{x\left(\;high\right)-x\left(low\right)}{2}}$ **, x: ω …, -3, -2, -1, 0, 1, 2, 3, …. +ω**				

**TABLE 2:** H_2_S gas adsorption experiments under different conditions by Sc-MOF@SiO_2_ core/shell nanostructures (design by 2^k−1^ factorial).

Run	Std order	Center Pt	A (bar)	B (min)	C (°C)	Rep	H_2_S adsorption (mmol/g)
a	2	1	0	0	+1	1	0.9
						2	0.9
b	4	1	−1	+1	+1	1	0.4
						2	0.3
c	3	1	−1	0	0	1	2.9
						2	2.8
d	1	1	+1	−1	−1	1	5.1
						2	5.0
e	5	0	0	0	0	1	3.4
						2	3.5

#### 3.2.2 Systematic study for H_2_S gas adsorption

**FIGURE 8:**
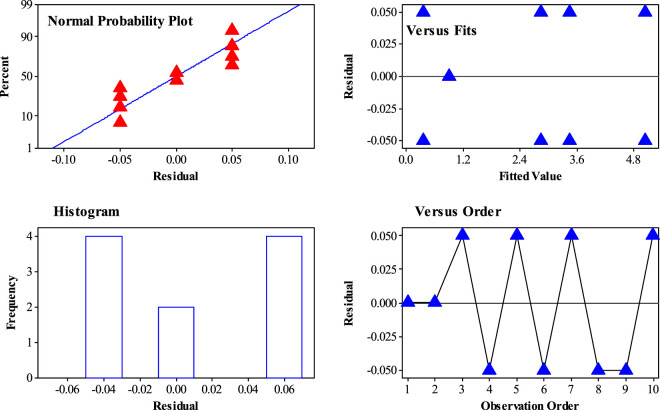
Different residual plot for H_2_S gas adsorption by Sc-MOF@SiO_2_ core shell nanostructures.

Figure 8 shows the residual plots for different distributions of the experiments in a 2^k−1^ factorial design. Based on the results, there is no evidence of nonrandom distribution of experiments in all kinds of experiments. As an important result, the randomized distribution of H_2_S gas adsorption was confirmed by a 2^k−1^ experimental design.

The results of the analysis of variance for H_2_S gas adsorption are shown in Table 3. Based on these results, the amount of P_value_ for all three factors (pressure, time contact, and temperature) is close to 0.000. This amount indicates the effective effect of experimental parameters on the efficiency of H_2_S gas adsorption.

**TABLE 3:** Analysis of variance for H_2_S gas adsorption (coded units).

Source	DF	Seq SS	Adj SS	Adj MS	F	P
Main effects	3	28.6494	26.5000	8.83333	1766.67	0.000
A	1	13.6406	0.3600	0.36000	72.00	0.001
B	1	15.0045	4.3350	4.33500	867.00	0.000
C	1	0.0043	0.1707	0.17067	34.13	0.004
2-Way interactions	2	0.7666	0.7666	0.38328	76.66	0.001
A*B	1	0.5959	0.0010	0.00100	0.20	0.048
B*C	1	0.1707	0.1707	0.17067	34.13	0.004

**FIGURE 9:**
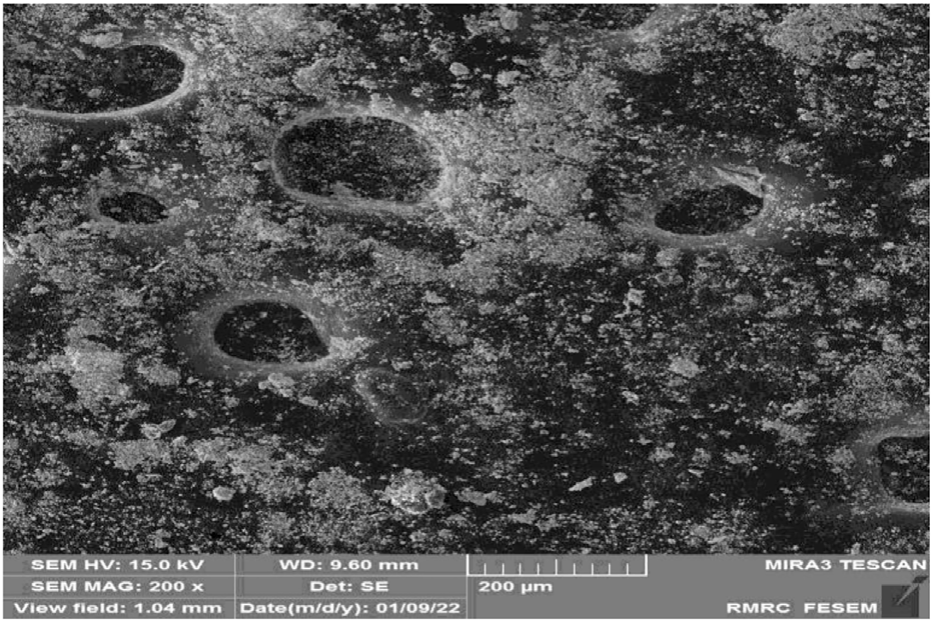
SEM images of Sc-MOF@SiO_2_ core/shell nanostructures in condition b (Table 2).

In fact, by increasing the contact time, the interaction between the adsorbent and H_2_S gas molecules can be increased. As shown in condition b (Table 2), this amount to some extent affects the amount of gas adsorption, and then the efficiency of the sample may decrease (Li et al., 2009). As an important result, the Sc-MOF nanostructures (core) and SiO_2_ powders (shell) may be agglomerated into each other if the contact time is high. To verify this, SEM images were taken of Sc-MOF@SiO_2_ core shell nanostructures in condition ***b*** which, as it turns out, the particles tended to agglomerate (Figure 9) (Guo et al., 2020). This problem can affect the efficiency of the nanostructure (0.4 mmol/g H_2_S gas adsorption in condition b compared to 5 mmol/g in optimal conditions). Other parameters affecting the rate of H_2_S gas adsorption include temperature. As the temperature increases, the effective collision level increases, and this affects the performance of the Sc-MOF@SiO_2_ core/shell nanostructures in H_2_S gas adsorption (condition b). The effect of pressure on H_2_S gas adsorption is also in accordance with previous studies (Xiao et al., 2009).

**FIGURE 10:**
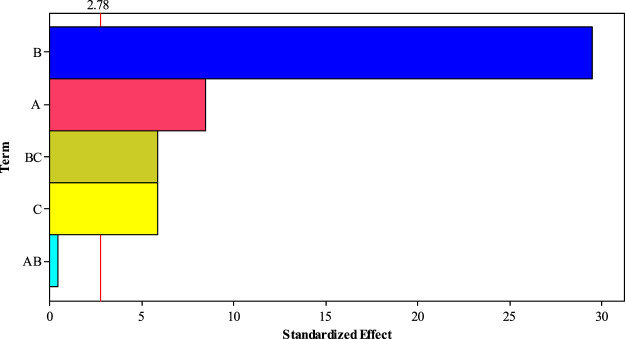
Pareto chart for different conditions of a 2^k−1^ design in H_2_S gas adsorption.

**FIGURE 11:**
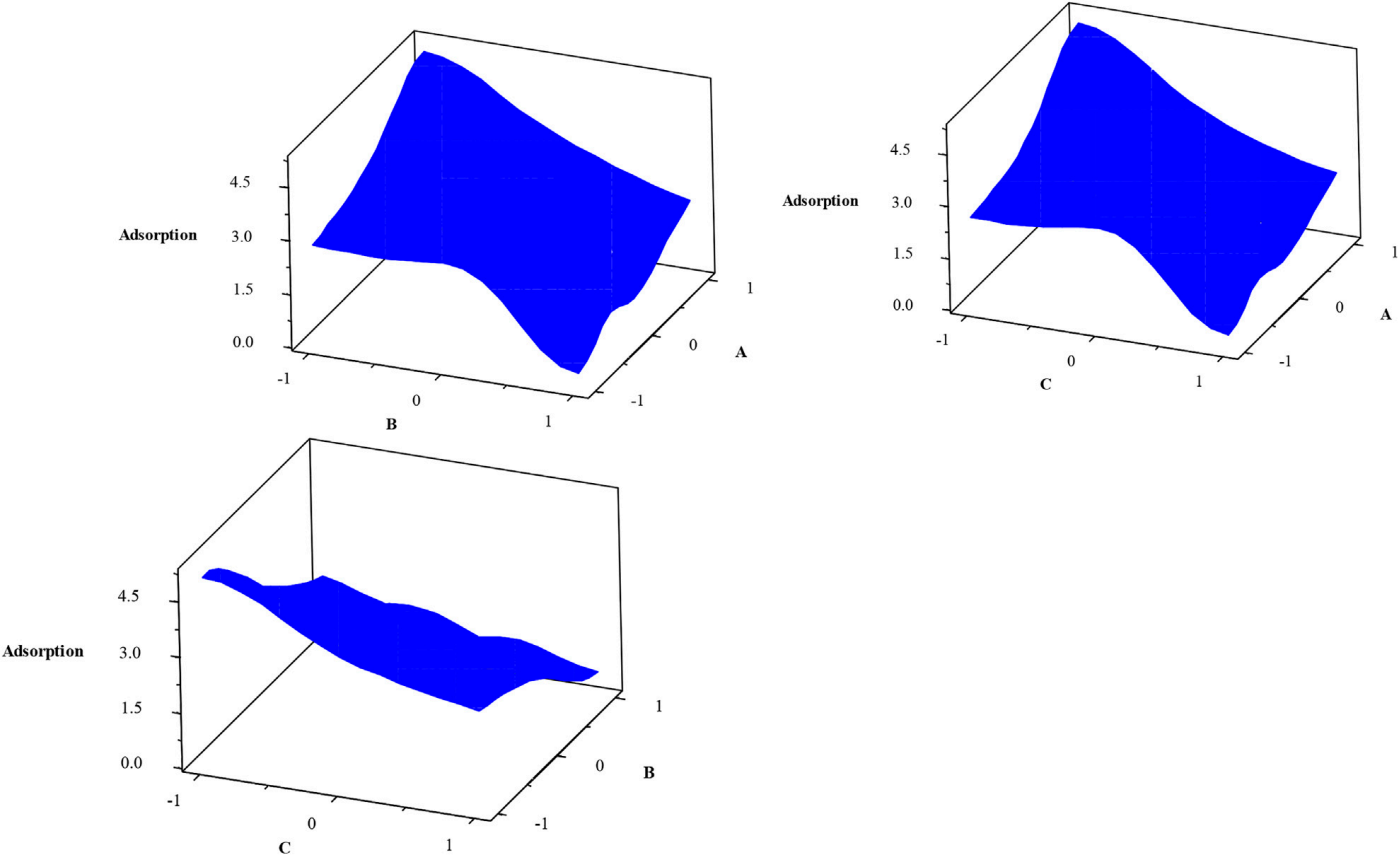
3D surface plot for the relation between experimental parameters and H_2_S gas adsorption.

The Pareto chart (Figure 10) also confirms the significant effects of experimental parameters (pressure, time contact, and temperature) on H_2_S gas adsorption. This figure also shows the high efficiency of H_2_S gas adsorption by the Sc-MOF@SiO_2_ core/shell nanostructures. The results of the Pareto chart agree with the data obtained from the analysis of variance, which confirmed the significant effects of pressure, time contact, and temperature on CH_4_ gas adsorption. The relationship between experimental parameters and gas absorption is very important. This relationship is schematically shown in Figure 11. As known, by selecting any of the values, the relevant answers can be obtained.

## 4 Conclusion

In this study, for the first time, Sc-MOF@SiO_2_ core/shell nanostructures were developed using novel ultrasonic-assisted microwave routes in mild conditions. These novel nanostructures showed distinctive properties such as high specific surface area (3,700 m^2^/g), significant porosity (more than 2 nm), narrow particle size distribution (less than 100 nm), and high thermal stability (328°C). The results of 2^K−1^ factorial experimental designs showed that the Sc-MOF@SiO_2_ core/shell nanostructures have an adsorption rate of 5 mmol/g in optimal conditions. It seems that the development of ultrasonic-assisted microwave routes and the introduction of new nanostructures with immobilization of Sc-MOF in the core–shell network may affect the functional efficiency of the Sc-MOF@SiO_2_ core/shell products.

## Data Availability

The raw data supporting the conclusions of this article will be made available by the authors, without undue reservation.
